# The Effects of Different Hormones on Supraventricular and Ventricular Premature Contractions in Healthy Premenopausal Women

**DOI:** 10.3390/medicina57111154

**Published:** 2021-10-24

**Authors:** Elias Tsougos, Emmanouil Korakas, Vasileios Kolovos, Georgia-Angeliki Koliou, Georgios Papageorgiou, Aikaterini Kountouri, Konstantinos Balampanis, Loukia Pliouta, Savvas Loizos, Nikolaos Karamichalakis, Elias Gatos, Ignatios Ikonomidis, Paraskevi Kazakou, Athanasios Raptis, Vaia Lambadiari

**Affiliations:** 1 Heart Failure and Preventive Cardiology Section, Department of Cardiology, Hygeia Hospital, 15123 Athens, Greece; ITsougos@hygeia.gr (E.T.); drkolovos@gmail.com (V.K.); loizos@kardiodiagnosi.gr (S.L.); karamichalakis@gmail.com (N.K.); 2 Second Department of Internal Medicine, Attikon University Hospital, Medical School, National and Kapodistrian University of Athens, 12462 Athens, Greece; mankor-th@hotmail.com (E.K.); gkoliou@buffalo.edu (G.-A.K.); KaterinaK90@hotmail.com (A.K.); kostasbalabanis@gmail.com (K.B.); plioutaloukia@gmail.com (L.P.); atraptis@med.uoa.gr (A.R.); 3 Independent Researcher, 10674 Athens, Greece; papahorhe@gmail.com; 4 Embio Medical Center, 15231 Athens, Greece; elias.gatos@gmail.com; 5 Second Cardiology Department, Attikon University Hospital, Medical School, National and Kapodistrian University of Athens, 12462 Athens, Greece; ignoik@gmail.com; 6 Department of Clinical Therapeutics, National and Kapodistrian University of Athens,11528 Athens, Greece; vkazakou@hotmail.com

**Keywords:** estradiol, progesterone, premature ventricular contractions, menstrual cycle, arrhythmias

## Abstract

*Background and Objectives*: The effects of gender differences on cardiac parameters have been well-established. In this study, we aimed to evaluate the possible associations of plasma levels of different sex hormones with premature atrial or ventricular contractions in premenopausal women. *Materials and Methods*: We conducted a prospective study which included women in late reproductive age who presented with palpitations during an eight-month period. A 12-lead electrocardiography, a transthoracic echocardiogram, blood samples, and 24-hour rhythm Holter were conducted on the third day of the menstrual cycle. *Results* Overall, 93 healthy premenopausal women with a median age of 42 years were enrolled. QTc interval was within normal limits in all patients. The 24 h range of premature atrial contractions (PACs) and premature ventricular contractions (PVCs) was 0–6450 and was 0–21,230, respectively. The median number of PVCs was 540 and the median number of PACs was 212, respectively. In total, 51 patients (54.8%) had a frequency of PVCs > 500/24 h and 37 patients (39.8%) had a frequency of PACs > 500/24 h, respectively. No statistically significant association was shown between any hormone and the frequency of PACs. Regarding PVCs, patients with a PVCs frequency > 500/24 h had higher estradiol levels compared to patients with PVCs less than 500/24 h (median 60 pg/mL versus 42 pg/mL, *p* = 0.02, OR: 1.01). No association was found between PVCs and other hormones. *Conclusions*: In premenopausal healthy women, higher estradiol levels are independently associated with increased PVCs. This suggests that estradiol in late reproductive stages may exert proarrhythmic effects.

## 1. Introduction

Gender differences in the incidence of cardiac arrhythmias have been well documented [1]. Beginning since puberty and persisting, even to a smaller extent, after menopause, women have higher resting heart rates and longer corrected QT intervals, while female gender has been established as a distinct risk factor for Torsades de Pointes, especially when receiving QT prolonging medication [2,3]. As a number of electrophysiological parameters have been shown to vary during the usual menstrual cycle, such differences in the electrocardiogram (ECG) values and susceptibility to arrhythmias are assumed to be related to sex hormones, such as estradiol (E2) and progesterone (PRG) [4]. Many studies have been conducted about the effect on sex hormones on QT interval duration, with E2 being generally considered to promote QTc lengthening and progesterone exerting the opposite action, although results have been inconclusive; on the contrary, little data is available about the effects of these hormones on specific arrythmias, such as premature ventricular contractions (PVCs) or premature atrial contractions (PACs) [5]. A significant amount of data is also available regarding the association between abnormal levels of thyroid stimulating hormone (TSH) and heart rate dysregulations [6], while data about glycosylated hemoglobin (HbA1c) or other pituitary hormones are scarce [7,8]. In this study, we aimed to investigate the possible association of different hormones with the frequency of PVCs or PACs in heathy premenopausal women who presented with palpitations.

## 2. Materials and Methods

### 2.1. Design and Study Population

The study was designed as an observational prospective study. We evaluated 93 consecutive, healthy, premenopausal female patients, age 40–46 years old, who presented to our practice during an eight-month period with palpitations over an eight-month period. All of the participants confirmed that they were in the reproductive age range and did not have a remarkable medical history nor were they under any medications at the time of the evaluation. Vital signs including heart rate, arterial hypertension, body temperature and oxygen saturation were checked. After a 10-minute resting period, a 12-lead resting electrocardiogram (ECG) (Marquette Mac 5500, GE Healthcare Bio-Sciences, Pittsburgh, PA, USA) was conducted at 50 mm/s and 20 mm/mV voltage in a supine position and revealed sinus rhythm with PR, QRS and heart rate-corrected QT intervals being within normal ranges. A transthoracic echocardiogram (Vivid T9, GE Healthcare Bio-Sciences, Pittsburgh, PA, USA) was performed for all patients to exclude structural heart disease.

The exclusion criteria were patients with other than sinus rhythm, a medical history of diabetes mellitus, hypertension, cardiovascular disorders, antiarrhythmic medication, abnormal thyroid function findings, exogenous hormonal administration including oral contraceptives, and refusal to provide written consent.

Each patient underwent 24 h Holter monitoring (SEER 1000 Holter recorder, GE Healthcare Bio-Sciences, Pittsburgh, PA, USA) on the same day the blood samples were obtained. A value of PVCs or PACs greater than 500/24 h was used as a cut-off of clinically significant arrhythmias, as previously described [9]. All patients were asked to continue their normal daily routine during the recording. All patients revealed sinus rhythm, premature atrial contractions (PACs), premature ventricular contractions (PVCs) or both. Hormone levels were measured on the 3rd day of the menstrual cycle. A hormonal panel including E2, PRG, follicle-stimulating hormone (FSH), luteinizing hormone (LH), prolactin (PRL), TSH and glycosylated hemoglobin (HbA1c) was obtained by all participants on the day of the examination.

This study was approved by the institutional ethical board of Hygeia Hospital (date: 9 February 2020, code number: 11330). A signed informed consent was obtained from all subjects prior to any procedure included in the study protocol. All methods were carried out in accordance with relevant guidelines and regulations (Declaration of Helsinki).

### 2.2. Statistical Analysis

IBM SPSS 23 software (IBM Corp., Armonk, NY, USA) was used for statistical analysis. Descriptive statistics including absolute and relative frequencies for categorical variables and medians with the respective minimum and maximum values for continuous were used to summarize patient characteristics and hormonal levels. The potential associations between the hormonal levels and PVCs or PACs (treated as continuous variables) were examined by spearman correlations. A value of PVCs or PACs greater than 500/24 h was used as a cut-off of clinically significant arrhythmias. The differences in hormonal levels between the groups of patients defined by the frequency of PVCs (>500 PVCs/24 h versus ≤500 PVCs/24 h) and PACs (>500 PACs /24 h versus ≤500 PACs /24 h) were assessed via the non-parametric Wilcoxon rank-sum test. Univariate logistic regression models were applied to evaluate the effect of hormonal levels on the presence of >500 PVCs/24 h and >500 PACs/24 h. Subsequently, variables that showed (marginal) statistical significance in univariate analysis were adjusted for weight and smoking status, parameters already known as significant risk factors. 

All tests were two-sided at a 5% level of significance.

## 3. Results

### 3.1. Demographic Characteristics

Overall, 93 consecutive patients presenting with palpitations were enrolled in the study. The median age was 42 (range 40–46) years, and the median weight was 62 (range 49–110) kilograms. In total, 22 patients (23.7%) had a history of smoking.

### 3.2. The Prevalence of Pvcs or Pacs among the Patients

The 24 h range of PACs was 0–6450 and that of PVCs was 0–21,230, respectively. The median number of PVCs and PACs was 540 and 212, respectively. Overall, 51 patients (54.8%) had a frequency of PVCs > 500/24 h and 37 patients (39.8%) had a frequency of PACs > 500/24 h, respectively, while 21 patients (22.6%) had a frequency of PACs > 500/24 h along with a frequency of PVCs > 500/24 h. The median number of PVCs among those with a frequency of PVCs > 500/24 h was 2450 (range 540–21,230) as opposed to a median number of 1340 PACs among patients with a frequency of PACs > 500/24 h (range 540–6450). 

### 3.3. Associations between Hormonal Levels and Cardiac Parameters

A significant, positive correlation was detected between E2 levels and PVCs (spearman rho = 0.22, *p* = 0.04), which, however, was not strong, whereas no further correlations were identified between hormone levels and PVCs or PACs. Additionally, patients with a PVCs frequency > 500/24 h had higher E2 levels compared to those with PVCs less than or equal to 500/24 h (median value 60 pg/mL versus 42 pg/mL, Wilcoxon rank-sum *p* = 0.02) with the odds of presenting PVCs > 500/24 h marginally significantly increasing with a one-unit increase in E2 levels (odds ratio (OR) = 1.01, 95% CI 1.00–1.02, Wald’s *p* = 0.052). No statistically significant association was shown between any hormone and the frequency of PACs. (Table 1 and Table 2). QT and corrected QT (QTc) interval (using the Bazett’s formula) were within normal range for all patients. Upon adjustment for weight and smoking status, E2 levels remained an independent significant predictor for PVCs > 500/24 h with the odds of appearance of >500 PVCs/24 h increasing by 2% (OR = 1.02, 95% CI 1.00–1.04, *p* = 0.02). Weight did not show predictive significance in the multivariate model (OR = 0.98, 95% CI 0.95–1.03, *p* = 0.45), while a trend towards increased odds of a frequency of >500 PVCs/24 h was observed for smoking (OR = 2.82, 95% CI 0.89–8.92, *p* = 0.08) in the multivariate analysis.

## 4. Discussion

This study revealed that, in the early stage of the follicular phase of the menstrual cycle, a positive association between E2 levels and PVC frequency was determined to exist. To our knowledge, this is the first report that shows a possible detrimental effect of E2 on PVCs in human subjects. In general, E2 has negative inotropic and cronotropic properties. In a study by Philp et al. [10], the acute intravenous administration of 17β-E2 in rat isolated ventricular monocytes under conditions of acute coronary artery occlusion significantly reduced the number of PVCs and the incidence of ventricular tachycardia, with this effect being attributed to the inhibition of the cardiovascular L-type Ca2+ current. Similar results were reproduced in another report by Ullrich et al. [11], while Chen et al. [12] proposed the increase of Connexin43, a gap junction protein in the ventricular myocardium, after chronic treatment with physiological concentrations of E2 as a potential antiarrhythmic mechanism in infarcted rats. Node et al. [13] reported that exogenous E2 administration reduces ventricular arrhythmias during ischemia in dogs. As, however, regulation of ion channels is species-specific, it is doubtful whether such mechanisms can be extrapolated into humans.

Regarding human studies, data is scarce (Table 3). In a study on women of reproductive age, Dogan et al. [14] showed that the frequency of premature ventricular contractions (PVCs) decreased significantly from menstruation to ovulation period (210 beats/day and 86 beats/day, respectively), an effect which was attributed to the E2 peak during ovulation. In favor of E2, Hu et al. [15] showed that E2 levels were lower in postmenopausal women with idiopathic outflow tract ventricular arrhythmias (IOTVA) compared to controls, and that after a three-month estrogen treatment, the ventricular arrhythmia count was significantly decreased (3958 ± 1972 vs. 10171 ± 6091 beats/24 h, respectively; *p* < 0.001). The reasons for the discrepancy with our results are not clear, however, possible assumptions can be made. One explanation might be the fact that, in our cohort, a single measurement was performed for each hormone, so it was not possible to determine the effect of hormonal fluctuations on cardiac rhythm during the menstrual cycle. In addition, recent data have implied that ECG parameters are not only determined by the absolute values of steroid sex hormones such as E2 and PRG. In a study by Abehsira et al. [16], PRG/E2 ratio and FSH levels were positively associated with QTc interval, implying that the relative concentrations of hormones and gonadotropin levels may also play an important role in ventricular repolarization and, maybe, arrhythmias. In addition, sex hormones influence the autonomic tone. Women seem to have a more dominant vagal tone [17], however, low levels of estrogen increase sympathetic activity in the luteal phase of the menstrual cycle [18]. Similarly, in post-menopausal women an increased predominant sympathetic tone is associated with reduced estrogen levels [19]. Lastly, the age range in our study (40–46 years) was quite different from the other two human studies mentioned, and it is known that the limits between the different stages of reproductive aging are often vague. In fact, according to the STRAW criteria, women at the age of 40 are on the verge of Stage-3, a period over which the ovarian reserve fluctuates; on the other hand, women at the age of 46 approach Stage-2, where the ovarian secretory pattern becomes highly erratic, or even Stage-1, namely late menopausal transition, where estrogen levels are more likely to be low, but they can also be unexpectedly high even during anovulatory cycles [20]. It becomes evident that at this life stage, hormonal levels and their effects on cardiac parameters are less predictable and may be different from their effects in earlier stages of reproductive life, especially as FSH levels also tend to vary, even in a manner which is independent from sex steroids levels.

In our study, the QTc interval was within normal limits in all participants. In general, data about the effect of E2 on QTc interval are inconclusive [5,21]. In premenopausal women before and after oophorectomy, De Leo et al. [22] failed to show any significant change in the QTc duration after surgery and, similarly, Saba et al. [23] failed to show any difference in QTc interval between premenopausal and postmenopausal women, despite the obviously lower E2 levels in the second group. On the contrary, a retrospective case-control study on 60 postmenopausal women showed increased QTc duration in women under estrogen replacement therapy compared to controls [24], and Kadish et al. [25] showed that women under estrogen-alone replacement therapy had longer QTc intervals compared to untreated women; however, this effect was not observed in women receiving estrogen-plus-PRG treatment. PRG seems to shorten QTc interval in a number of studies. In two different prospective interventional studies [26,27], double autonomic blockade with atropine and propranolol revealed a shorter QTc interval in the luteal phase of the menstrual cycle, where PRG reaches its highest levels, and a similar result was shown by Nakagawa et al. [28] regardless of any differences in the PR intervals. In our study, the QTc interval was not evaluated in different phases of the menstrual cycle, so any effects of sex hormones on its duration cannot be made clear. It must be noted again that the age range in the aforementioned studies significantly differs from ours, and the same applies to the sample size, too. Regarding PACs, the absence of any association with hormonal levels in women is confirmed by the available literature, which indicates, however, a higher susceptibility to supraventricular tachycardia (SVT), atrioventricular nodal re-entry tachycardia (AVNRT), and atrial fibrillation (AF) in a possibly hormone-mediated mechanism [29].

**Table 3:** Major studies on the association between hormone levels and electrocardiographic changes.

Study	Population	Measurements	Results
Dogan et al. [14]	20 premenopausal women with PVCs—18 healthy controls	1st or 2nd day of menstruation—repeat at ovulation period	Negative association between PVCs and E2 levels—No association between PVCs and PRG
Hu et al. [15]	35 postmenopausal women with IOTVA under ORT—35 controls	Single measurement of plasma sex hormones	Lower E2 levels in patients with IOTVA—No association for LH, progestogen
Abehsira et al. [16]	84 CAH patients—84 healthy controls	Single measurement of plasma sex hormones	Negative association between QTcF and PRG/E2 ratio in women—Positive association with FSH in men
De Leo [22]	41 women with hysterectomy and ovariectomy (26 premenopausal—15 postmenopausal)	3 measurements of E2 and FSH: admission (all), after surgery (all), after randomization for transdermal ethinyl estradiol (premenopausal women)	No association between hormones and QTc
Saba et al. [23]	101 women (36 premenopausal—65 postmenopausal, of whom 15 on HRT)	Single ECG	No association between QTc and estrogen
Haseroth et al. [24]	60 postmenopausal women (16 ERT, 22 PERT, 22 controls)	Single ECG	Lower QTc with PERT—higher QTc with ERT
Kadish et al. [25]	12,451 untreated women vs 21,927 women under hormone treatment	Single ECG	Positive association between estrogen and QTc—negative association for PRG
Burke et al. [26]	23 women—20 men	ECG before and after autonomic blockade	Shorter QTc in luteal phase (↑ PRG)
Entres et al. [27]	22 premenopausal women	ECG during menses, follicular and luteal phase at baseline and after autonomic blockade	Shorter QTc in luteal phase (↑ PRG)
Nakagawa et al. [28]	11 premenopausal women	Holter ECG during follicular and luteal phaseSerum catecholamine levels	Shorter QTc in luteal phase (↑ PRG)

PVCs: premature ventricular contractions; E2: estradiol; PRG: progesterone; IOTVA: idiopathic outflow tract ventricular arrhythmias; ORT: oestrogen replacement therapy; LH: luteinizing hormone; CAH: congenital adrenal hyperplasia; FSH: follicle-stimulating hormone; ERT: estrogen replacement treatment; PERT: progestin-estrogen replacement treatment; ECG: electrocardiogram.

No association was found between TSH levels and premature contractions, a result that was expected since only patients with normal TSH levels were enrolled. Apart from the well-established association between hyperthyroidism and atrial fibrillation, PACs have also been related to hyperthyroidism, whereas data about PVCs are limited and ventricular irritability has been linked mainly to hypothyroidism in several case reports [6,30,31]. In the same notion, the normal values of HbA1c in our cohort were not related to arrhythmias; high values of HbA1c in diabetes have been associated mainly with atrial fibrillation, apart from their already known role in the prediction of cardiovascular disease, together with other hematological and coagulation parameters [7,32,33]. However, cardiac autonomic neuropathy, which is mainly characterized by the generation of cardiac arrhythmias, is actually considered a form of diabetic neuropathy and involves a predominance of the sympathetic system and a concurrent damage to the parasympathetic system, which can eventually lead to hypertension and macrovascular complications [34]. Lastly, as expected, no relation of PRL to arrhythmias was shown; in fact, the only association between PRL and the heart exists in the pathogenesis of peripartum cardiomyopathy, where a small antiangiogenic subfragment, 16-kDa prolactin, may provoke the disease by inducing endothelial damage [8].

The most important strength of our study was the relatively large sample size compared to other human studies on the field. In addition, it was conducted on women in their late reproductive stages, an age range which has not been addressed thoroughly in the majority of the previous studies. On the contrary, no cardiac MRI data were available to exclude any potential cardiac structural myocardial modification that could be associated with arrhythmias. Another serious limitation was the absence of multiple hormonal measurements in different phases of the menstrual cycle, which prevented more robust and detailed evaluation of the effect of various hormonal levels on cardiac parameters. However, the fact that the patients had no other medical history and that all hormones measured were within normal levels allows for any associations shown to be attributed to sex hormones with a higher degree of certainty.

## 5. Conclusions

In premenopausal healthy women with palpitations, higher E2 levels are independently associated with an increased frequency of PVCs. This suggests that, contrary to the previous data, E2 in late reproductive stages may exert proarrhythmic effects. More large-scale studies are needed to clarify the complex pathophysiological effects of sex hormones on cardiac parameters in this particular age group.

## Figures and Tables

**Table 1:** Hormone values and patient characteristics by PVC and PAC frequency.

		PVCs			PACs		
Variable	Total (N = 93)	≤500 PVCs/24 h (N = 42)	>500 PVCs/24 h (N = 51)	*p*-Value	≤500 PACs/24 h (N = 56)	>500 PACs/24 h (N = 37)	*p*-Value
Age (years)	42.0 (40.0, 46.0)	42.0 (40.0, 46.0)	42.0 (40.0, 45.0)	0.89	42.0 (40.0, 46.0)	42.0 (40.0, 45.0)	0.10
Weight (kilograms)	62.0 (49.0, 110.0)	64.0 (50.0, 110.0)	60.0 (49.0, 90.0)	0.18	63.0 (49.0, 90.0)	62.0 (49.0, 110.0)	0.61
FSH (mIU/mL)	8.0 (0.40, 78.4)	7.5 (1.3, 78.4)	8.1 (0.40, 41.6)	0.35	7.7 (0.40, 78.4)	8.9 (2.0, 42.9)	0.56
LH (IU/L)	5.9 (0.10, 64.7)	5.3 (1.01, 64.7)	6.3 (0.10, 41.7)	0.24	5.5 (0.10, 64.7)	6.4 (2.7, 43.7)	0.39
E2 (pg/mL)	51.0 (9.0, 351.0)	42.0 (9.0, 215.0)	60.0 (18.6, 351.0)	0.02	51.0 (13.0, 351.0)	51.0 (9.0, 225.0)	0.55
PRL (ng/mL)	17.0 (4.1, 70.0)	16.9 (4.1, 43.0)	17.0 (5.7, 70.0)	0.76	17.0 (5.7, 70.0)	16.7 (4.1, 48.7)	0.66
PRG (ng/mL)	0.40 (0.05, 11.0)	0.50 (0.05, 11.0)	0.40 (0.10, 9.5)	0.52	0.40 (0.05, 11.0)	0.40 (0.10, 9.0)	0.27
TSH (mIU/L)	1.7 (0.01, 30.2)	2.0 (0.01, 30.2)	1.7 (0.10, 3.6)	0.47	1.7 (0.03, 10.0)	1.5 (0.01, 30.2)	0.83
HbA1c (%)	5.2 (4.5, 6.0)	5.1 (4.5, 6.0)	5.3 (4.6, 5.9)	0.42	5.3 (4.5, 6.0)	5.2 (4.8, 5.8)	0.71

Values presented as median (min, max); PVCs: premature ventricular contractions; PACs: premature atrial contractions; FSH: follicle-stimulating hormone; LH: luteinizing hormone; E2: estradiol; PRL: prolactin; PRG: progesterone; TSH: thyroid-stimulating hormone; HbA1c: glycosylated hemoglobin.

**Table 2:** Odds ratios and 95% confidence intervals estimated by univariate logistic regression for the appearance of >500 PVCs/24 h and >500 PACS/24 h.

	>500 PVCs/24 h		>500 PACs/24 h	
Parameter	OR (95% CI)	*p*-Value	OR (95% CI)	*p*-Value
Age (years)	0.98 (0.77–1.23)	0.84	1.21 (0.95–1.54)	0.12
Weight (kilograms)	0.97 (0.94–1.01)	0.17	1.02 (0.98–1.06)	0.36
FSH	1.00 (0.96–1.03)	0.85	1.01 (0.97–1.05)	0.57
LH	0.99 (0.95–1.03)	0.71	1.01 (0.97–1.05)	0.81
E2	1.01 (1.00–1.02)	0.05	1.00 (0.99–1.01)	0.72
PRL	1.01 (0.97–1.05)	0.51	0.99 (0.95–1.03)	0.67
PRG	0.97 (0.78–1.20)	0.78	0.90 (0.69–1.17)	0.42
TSH	0.84 (0.63–1.11)	0.21	1.08 (0.94–1.24)	0.27
HbA1c	1.74 (0.43–6.98)	0.43	0.73 (0.18–2.95)	0.66

OR: odds ratio; CI: confidence interval; FSH: follicle-stimulating hormone; LH: luteinizing hormone; E2: estradiol; PRL: prolactin; PRG: progesterone; TSH: thyroid-stimulating hormone; HbA1c: glycosylated hemoglobin.

## Data Availability

The datasets used and/or analyzed during the current study are available from the corresponding author on reasonable request.
